# Immune responses to porcine epidemic diarrhea virus (PEDV) in swine and protection against subsequent infection

**DOI:** 10.1371/journal.pone.0231723

**Published:** 2020-04-28

**Authors:** Venkatramana D. Krishna, Yonghyan Kim, My Yang, Fabio Vannucci, Thomas Molitor, Montserrat Torremorell, Maxim C.-J. Cheeran

**Affiliations:** Department of Veterinary Population Medicine, College of Veterinary Medicine, University of Minnesota, St. Paul, Minnesota, United States of America; New York Blood Center, UNITED STATES

## Abstract

Understanding the immune responses against Porcine epidemic diarrhea virus (PEDV) is important to prevent infection and to design control strategies. We evaluated both systemic and mucosal immune responses to PEDV in pigs and assessed if prior exposure to virus protects against re-infection. Three-week-old pigs were infected with PEDV and immune response in blood, intestine, and mesenteric lymph node (MLN) was evaluated. At 30 dpi, virus exposed pigs were challenged with a field isolate of PEDV and immune response at 5 d post challenge was evaluated. We found that PEDV RNA persists in the intestine even after fecal shedding of the virus was stopped at 28 dpi and pigs previously exposed to PEDV are protected from virus shedding after re-infection. PEDV infection induced both humoral and cell mediated immune response with an increase in PEDV specific IgA and IgG antibodies in intestine and serum. Flow cytometry analysis showed a significantly higher frequency of B cells and lower frequency of T cells at 4 dpi. The frequency of CD4/CD8 double positive (DP) memory T cells was significantly increased in the MLN of challenged animals. These studies may provide further insights into understanding the mucosal immune response to PEDV and its role in protection against disease.

## Introduction

Porcine epidemic diarrhea virus (PEDV) is an enveloped single stranded positive sense RNA virus of the genus *Alphacoronavirus* in the family *Coronaviridae*. The virus is highly contagious and causes a devastating enteric disease in swine characterized by severe watery diarrhea and vomiting. Although the virus infects pigs of all ages, severe clinical disease and mortality is reported predominantly in neonatal suckling piglets [1]. Since its first identification in the United Kingdom and other European countries in the 1970s [2, 3], PEDV outbreaks have been reported in many pig producing countries including Japan, Korea, China and the United States (US). In Asia, PEDV outbreaks first occurred in Japan in 1982 and later spread to China and South Korea. Use of inactivated virus vaccines helped control PEDV in the region until early 2000’s, when a new variant of PEDV emerged in China, affecting vaccinated herds [4]. PEDV emerged in a completely naïve US swine population [5, 6] and rapidly spread across the country in 2013, reaching into Canada and Mexico resulting in significant economic losses for the swine industry due to increased mortality and decreased production.

PEDV mainly infects epithelial cells of the small intestine and transmission occurs predominantly by fecal-oral route through direct or indirect contact with infected pigs or feces contaminated material [7, 8]. The establishment of both systemic and mucosal immune responses, including secretion of virus specific IgA is essential to provide protection against PEDV infection [9–11]. Recently, it has been shown in a study using neonatal pigs that route of exposure influences immune response to PEDV infection, with oral infection inducing higher IgA titers both in serum and mucosa compared to intranasal or intramuscular inoculation [12]. In addition to antibody response, cellular immune responses may play an essential role in the protection against PEDV. Exposure to PEDV, through the feedback of fecal and/or homogenized intestine from acutely infected suckling piglets, is considered an important approach to develop short term immunity and to reduce morbidity and pre-weaning mortality in swine herds [6, 7, 13]. However, this feedback method has a potential risk to transmit other pathogens such as porcine reproductive and respiratory syndrome virus (PRRSV) and porcine circovirus type 2 (PCV 2), which may cause disease. Currently, modified live attenuated and inactivated vaccines are being used to control PEDV in the US. However these vaccines are not completely effective in providing protection and studies have shown that not all vaccinated sows develop protective lactogenic immunity [14]. The inconsistency in generating lactogenic immunity was attributed to the differences in immunization routes. Higher degree of protection with reduced mortality of suckling piglets was observed when pregnant sows were vaccinated orally with live attenuated PEDV compared to vaccination by intramuscular injection [15]. Although vaccination of sows with live attenuated PEDV reduced mortality of suckling piglets, they neither reduced the severity of diarrhea nor prevented virus shedding after challenge. Genetic differences between vaccine and field strains has been implicated as a possible reason for vaccine failure.

The objective of the present study was to evaluate both humoral and cellular immune responses to PEDV in infected pigs and to assess their role in protection against re-infection. Three-week-old pigs were infected with PEDV and the immune response to the virus was studied. At 30 day post infection (dpi), virus exposed pigs were challenged with a field isolate of PEDV to study the effectiveness of immune response generated by the initial exposure.

## Materials and methods

### Ethics statement

All experiments in this study using animals were conducted under protocols approved by the University of Minnesota Institutional Animal Care and Use Committee and in accordance with the Guide for the Care and Use of Laboratory Animals.

### Animals and housing

3-week-old cross bread pigs seronegative for PEDV were obtained from a commercial farm with no history of PEDV infection. Animals were housed in the isolation facility at the University of Minnesota. After arrival, rectal swab samples collected from pigs were tested for PEDV, transmissible gastroenteritis virus (TGEV), and porcine delta corona virus (PDCoV) by real-time reverse transcription polymerase chain reaction (rRT-PCR) at the University of Minnesota Veterinary Diagnostic Laboratory. Experimental animals were monitored twice daily for adverse signs.

### Cells and viruses

Vero 76 cells (ATCC CRL-1587, Manassas, VA, USA) were maintained in Dulbecco’s modified Eagle medium (DMEM) supplemented with 5% heat inactivated fetal bovine serum (FBS). PEDV strain USA/Colorado/2013 (GenBank accession number KF272920) was obtained from the National Veterinary Services Laboratory in Ames, IA and propagated in Vero 76 cells in DMEM supplemented with 0.5 μg/mL TPCK-trypsin (Worthington Biochemical Corporation, Lakewood, NJ, USA) and 0.3% tryptose phosphate broth (Sigma, St. Louis, MO, USA). Virus was purified from the clarified cell culture supernatants by ultracentrifugation through a 20% (w/v) sucrose cushion. Briefly, culture supernatant from PEDV USA/Colorado/2013 infected Vero 76 cells were clarified by centrifugation at 3000 x g for 30 min at 4 ^o^C to remove cell debris. Clarified culture supernatant was overlayed on a cushion of 20% buffered sucrose and centrifuged for 2 h at 112,600 x g, 4°C. The pellet was resuspended in sterile PBS and stored in aliquots at -80^o^ C. Culture supernatant from uninfected Vero 76 cells were processed similarly to use for mock control antigen. For use as antigen in ELISA, purified virus was heat inactivated at 60 ^o^C in a water bath for 1 h. Concentration of total protein in heat inactivated virus stock was determined using Pierce^™^ BCA protein assay kit (Thermo Scientific, Rockford, IL, USA) according to manufacturer’s instructions.

### Infection of pigs with PEDV

Three-week-old pigs (n = 12) were inoculated via intragastric route with intestinal mucosal scraping from animals infected with PEDV USA/Colorado/2013. Each pig was infected with 10 mL of virus inoculum containing 3.6×10^4^ 50% tissue culture infective dose per mL (TCID_50_/mL). Blood was collected at 4, 7, 10, and 21 dpi, and intestinal tissue and mesenteric lymph node (MLN) were collected at 4, 10, and 21 dpi to evaluate immune responses.

### Exposure of pigs to PEDV and viral challenge

To study if prior exposure to PEDV protects against re-infection, three-week-old pigs (n = 10) in a low biosecurity animal room were experimentally exposed to PEDV by direct contact with personnel working in an infected animal room as described previously [8]. Briefly, study personnel were allowed to interact directly with seeder pigs experimentally infected with PEDV (USA/Colorado/2013) by intragastric route in an infected animal room. Following 45 minutes interaction with infected seeder pigs, personnel entered low biosecurity sentinel holding room without changing their personal protective equipment (PPE) and interacted with pigs in the low biosecurity room for 45 minutes to allow for transmission of virus to the pigs in low biosecurity room. This procedure was repeated every day until all the pigs in low biosecurity room tested positive for PEDV. Rectal swab samples were collected daily to assess virus shedding. At 30 dpi, PEDV exposed pigs in the low biosecurity room were randomly divided into 2 groups (n = 5 in each group) and housed in two separate rooms. One group of PEDV exposed pigs was challenged via oral route with intestinal mucosal scrapings obtained from acutely infected animals from field strain of PEDV and the other group of PEDV exposed animals was left without challenge. The mucosal scrapings were confirmed to have a high PEDV load, using a quantitative RT-PCR assay. A third group of control naïve age matched pigs (n = 4) were challenged with the same field isolate of PEDV, to compare challenged animals with those getting primary exposure to the virus.

### Rectal swab samples

Rectal swab samples were collected daily using BBL^TM^ CultureSwab^TM^ collection and transport system (Becton Dickinson and Co, Sparks, MD). Each swab was suspended in 2 ml of DMEM supplemented with 2% BSA, 1% antibiotic-antimycotic, 0.15% TPCK-trypsin, and 0.1% gentamicin and stored at -80°C. 50 μl of this suspension was used to extract RNA.

### Intestinal homogenate

Proximal part of ileum was collected and rinsed with Ca^++^ and Mg^++^ free Hank’s balanced salt solution (HBSS). After measuring the weight, approximately 1g of tissue was cut into small pieces and homogenized using MagNA Lyser (Roche Diagnostics GmbH, Germany), 3 runs of 30 sec at 5,000 rpm. Homogenate was centrifuged at 10,000 x g for 10 min at 4°C and the supernatant was used for RNA extraction and ELISA.

### PEDV quantitative real time RT-PCR

Levels of PEDV RNA in rectal swab and intestinal homogenate samples were quantified by real time RT-PCR. RNA was extracted from 50 μl of the sample using MagMAX^^™^^ 96 viral RNA isolation kit (Thermo Fisher Scientific, Waltham, MA, USA), according to the manufacturer’s instructions. The purity of RNA was assessed by calculating the ratio of OD_260_/OD_280_. A portion of the PEDV S gene was amplified with the following primer pairs: Forward 1910: ACGTCCCTTTACTTTCAATTCACA and Reverse 2012: TATACTTGG TACACACATCCAGA- GTCA. PCR amplification was quantified using a FAM labeled probe 1939: FAM-TGAGTTGAT-TACTGGCACGCCTAA ACCAC-BHQ. The primer and hydrolysis probe set were added to the AgPath-ID^^™^^ One-Step RT-PCR Reagents (Thermo Fisher Scientific, Waltham, MA, USA) along with 5 μl of extracted total RNA and amplified using Applied Biosystems^™^ 7500 Fast Real-Time PCR System (Thermo Fisher Scientific, Waltham, MA, USA). Cycling conditions used were as follows: reverse transcription at 45°C for 10 min; denaturation at 95°C for 10 min; 45 cycles of denaturation at 95°C for 15 s and annealing at 60°C for 45 s. Viral RNA levels were normalized to 1 gram of intestinal tissue.

### PEDV ELISA

PEDV specific IgG and IgA in intestinal homogenate and serum were determined by PEDV antibody ELISA optimized in the laboratory. Microtiter plates (Corning Inc, Corning, NY) were coated with 100μl of 10μg/ml heat inactivated purified PEDV USA/Colorado/2013 in phosphate buffered saline (PBS). After overnight incubation at 4°C, the wells were blocked with 1% bovine serum albumin (BSA) and 1% normal goat serum in PBS for 1 h at room temperature. 100μl of diluted serum or intestinal homogenate was then added to duplicate wells and incubated for 1 h at room temperature. Serum samples were diluted 1:125, 1:250, 1: 500, 1:1000, and 1:2000 and intestinal homogenate samples were diluted 1:8, 1:16, 1:32, 1:64, 1:128. Wells were washed five times with wash buffer (PBS with 0.05% Triton X-100) and incubated for 30 min at room temperature with 100μl of horseradish peroxidase (HRP) conjugated goat anti-pig IgG (Jackson ImmunoResearch, West Grove, PA; 1:3000 diluted) or goat anti-pig IgA (Bethyl Laboratories, Montgomery, TX; 1:10000 diluted). After washing the wells five times with wash buffer, 100μl of 1 step ultra TMB (Thermo scientific, Rockford, IL) was added and the reaction was stopped after 30 min incubation at room temperature by adding 100μl of 1N H_2_SO_4_. The absorbance at 450nm was measured using a microplate reader (Synergy H1, BioTek Instruments, Inc). The cut off value was calculated as mean of negative control multiplied by 2.

### PEDV neutralization assay

PEDV neutralizing antibody titers in serum and intestinal homogenate of pigs were determined by immunoplaque assay [16]. Briefly, samples were heat inactivated at 56°C for 30 min, serially diluted two-fold in DMEM containing 1 μg/ml TPCK trypsin and incubated with 200 TCID_50_ of PEDV for 1h at 37°C in a total volume of 500 μl. After incubation, serum-virus mixtures (250μl) were transferred to confluent Vero 76 cell monolayer in duplicate wells of 24-well tissue culture plate. Plates were incubated for 1h at 37°C before removing the inoculum and replacing with infection medium containing 1% low melting point (LMP) agarose. At 72 h p.i., the medium was removed from the wells and the cells were fixed with 4% paraformaldehyde for 20 min at 4°C. After three washes and blocking for 1h in PBS containing 5% normal goat serum and 0.3% triton X-100, plates were incubated overnight at 4°C with monoclonal antibody to PEDV Spike protein (Clone S1D12, VMRD, Pullman, WA; 1:500 diluted). Wells were then washed three times with wash buffer and incubated for 1 h at RT with alkaline phosphatase conjugated anti-mouse IgG secondary antibody (Thermo Fisher Scientific, Waltham, MA, USA; 1:200 diluted). Plates were washed three times and incubated for 20 min at RT with 1-Step^^™^^ NBT/BCIP substrate solution (Thermo Fisher Scientific, Waltham, MA). Immunostained cells were observed under a light microscope (Nikon, Tokyo, Japan). 50% plaque reduction neutralization titer (PRNT_50_) was determined as reciprocal of the highest dilution of serum that showed ≥50% neutralizing activity.

### Flow cytometry

Frequency of B lymphocytes and T lymphocyte sub-populations in peripheral blood mononuclear cells (PBMC) and MLN were determined by flow cytometry. PBMC were isolated by density gradient centrifugation using lymphocyte separation medium (Corning, Manassas, VA). Cells collected from the interphase were washed twice with phosphate buffered saline (PBS) and suspended in RPMI 1640 supplemented with 2% FBS. Cells from MLN were prepared by teasing apart the tissue and passing the cell suspension through 70μm cell strainer. Cells were washed twice with RPMI 1640 supplemented with 2% FBS and immunostained with panel of antibodies to identify B lymphocytes (CD21-APC, CD72a-PE, CD3-PECy7) or T lymphocytes (CD3-PECy7, CD4a-PerCPCy5.5, CD8a-FITC, TCR δ-chain-APC). Cells were acquired using FACSCanto flow cytometer (BD Biosciences) and data were analyzed using FlowJo software.

### Statistical analysis

Results are presented as mean ± SEM. One-Way Analysis of Variance (ANOVA) with Welch’s correction was used to determine the significance of the difference between data sets. A “p” value of less than 0.05 was considered significant. Statistical analyses were performed using Prism 7 software (GraphPad, La Jolla, CA)

## Results

### Prior exposure to PEDV prevents virus shedding following re-infection

**Fig 1:**
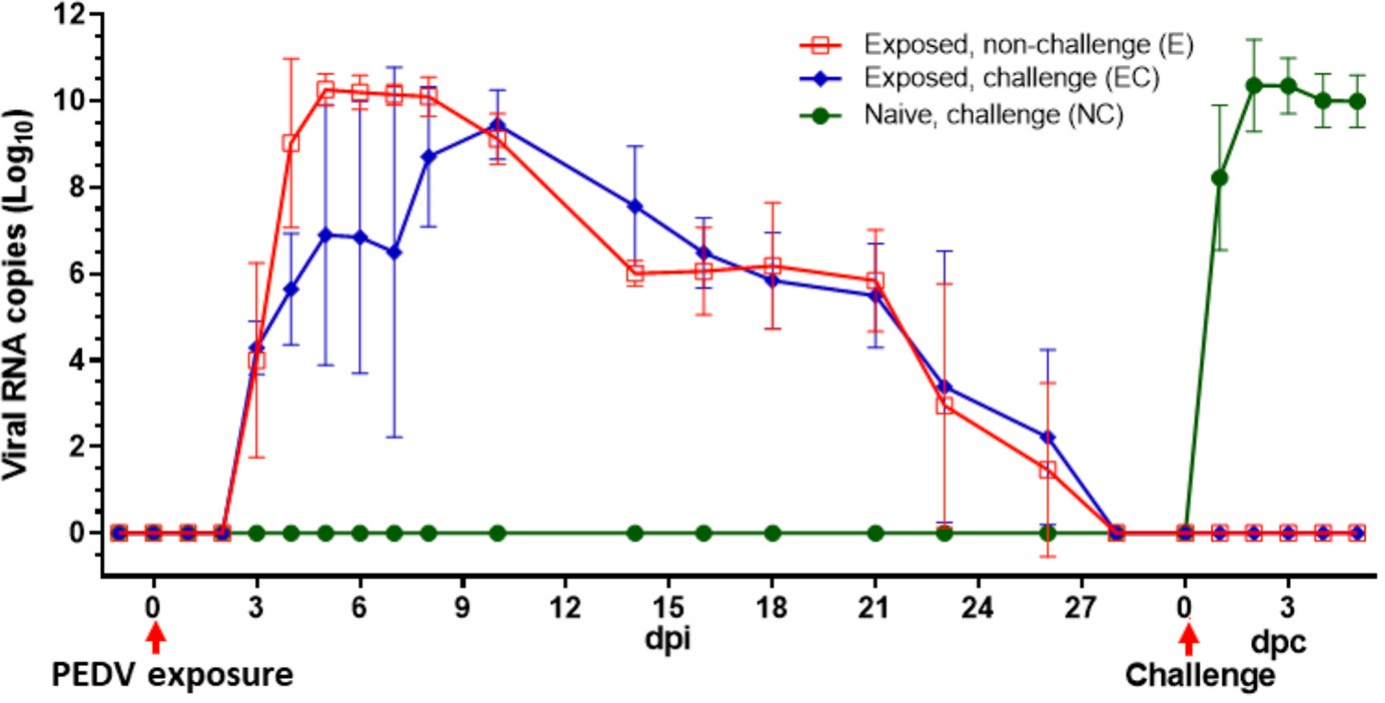
Prior exposure to PEDV prevents virus shedding upon subsequent infection. Three-week-old pigs were exposed to PEDV by contact with personnel working in infected animal room. At 30 dpi PEDV exposed pigs were challenged with field isolate of PEDV (group EC) or left without challenge (group E). As a control, age matched naïve pigs were challenged with the virus (group NC). Virus shedding was determined by RT-qPCR of rectal swab samples. (n = 3 to 5 in each group). dpi- day post infection; dpc- day post challenge.

Only 50% of the pigs exposed to PEDV USA/Colorado/2013 by contact with contaminated fomites showed signs of mild and transient diarrhea. However, on examination of rectal swab samples by RT-qPCR, viral RNA was detected in all pigs exposed to PEDV (Fig 1). PEDV RNA was detected in fecal material as early as 3 d post exposure and infected pigs continued to shed virus up to 26 dpi. Fecal swab samples from uninfected pigs were negative for PEDV RNA. PEDV RNA was not detected in rectal swab samples after 28 d post exposure. To determine if prior exposure to virus protects pigs from re-infection, a randomly selected group of PEDV exposed pigs were challenged orally with virulent field isolate of PEDV at 30 dpi. After challenge, viral RNA was detected in the rectal swab from all naïve control pigs (with no previous PEDV exposure) as early as 1 d post challenge (dpc). In contrast, viral RNA was not detected in the rectal swab samples in any of the challenged pigs with prior exposure to PEDV, suggesting that PEDV exposure protected against virus shedding in challenged pigs.

### PEDV RNA in the intestine

**Fig 2:**
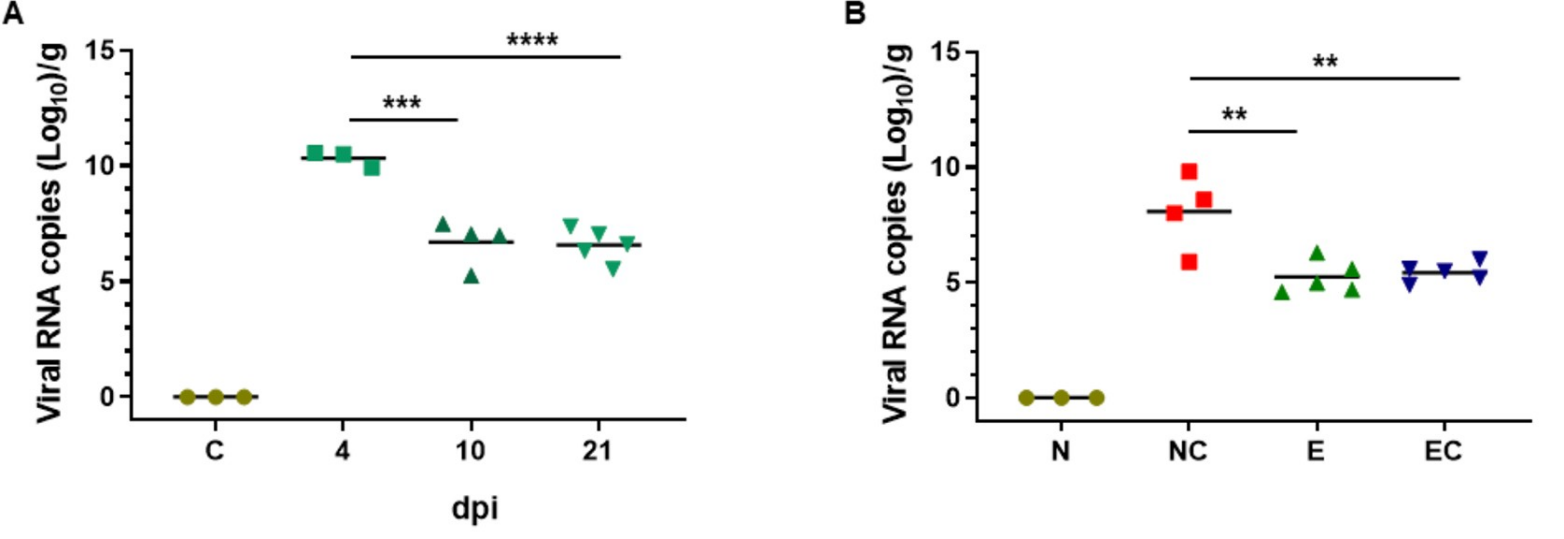
Intestinal viral RNA levels determined by RT-qPCR. (A) Three-week-old pigs were exposed to PEDV via intragastric route. Intestinal tissues were harvested at indicated dpi for analysis of viral RNA levels. (B) Three-week-old pigs were exposed to PEDV by contact with personnel working in infected animal room. At 30 dpi, PEDV exposed pigs were challenged with field isolate of PEDV. As a control, age matched naïve pigs were challenged with the same field isolate of virus. 5 days post challenge tissues were harvested for analysis. Viral RNA levels were normalized to one gram of intestinal tissue. N: naïve; NC: naïve, challenged; E: PEDV exposed; EC: PEDV exposed, challenged. **p<0.005, ***p<0.0005, and ****p<0.0001. (n = 3 to 5 pigs in each group).

Viral RNA load in intestinal tissue was measured at 4, 10, and 21 dpi from intestinal homogenate samples using RT-qPCR. Viral RNA was detected in the intestine of PEDV infected pigs at all the time points tested with peak viral RNA load detected at 4 dpi (Fig 2A). By 10 dpi, viral RNA levels were significantly reduced in the intestine by 4-log although further reduction in viral RNA levels was not observed at 21 dpi. To determine if prior exposure to PEDV protected against re-infection, PEDV exposed animals were challenged with field isolate of PEDV at 30 dpi. When tested 5 d after challenge, intestinal tissue of all the pigs with no previous PEDV exposure contained high concentration (mean 8.075 log_10_ copies/g) of viral RNA (Fig 2B). PEDV RNA was not detected in any of the non-challenged control pigs. Surprisingly, PEDV RNA ranging from 4.6 to 6.3 log_10_ copies per gram of tissue was observed in the intestine of PEDV exposed animals without challenge (35 dpi, Fig 2B “E”) at the time when no fecal shedding was detected by PCR in these animals. In addition, there was no significant increase in viral RNA load after challenge in pigs with prior exposure to PEDV, suggesting that PEDV RNA persists in the intestine even after fecal viral shedding had stopped. To confirm that the RNA detected in the intestine by RT-qPCR was specific to PEDV, we sequenced the PCR product and analyzed by BLAST (http://blast.ncbi.nlm.nih.gov). The result showed that all sequences had 100% similarity to PEDV spike protein gene, although this method could not distinguish PEDV strains used for the initial exposure from challenge virus.

### Antibody response in PEDV infected pigs

PEDV specific IgA and IgG antibodies in serum and intestinal homogenates were analyzed by ELISA using whole purified virus as antigen. Specificity of this ELISA was validated using serum samples from known PEDV infected and uninfected pigs (S1A and S1B Fig). Binding of PEDV antibody to the antigen coated wells was further confirmed by using commercially available monoclonal antibody to PEDV Spike protein (Clone S1D12, VMRD, Pullman, WA) (S1C Fig).

**Fig 3:**
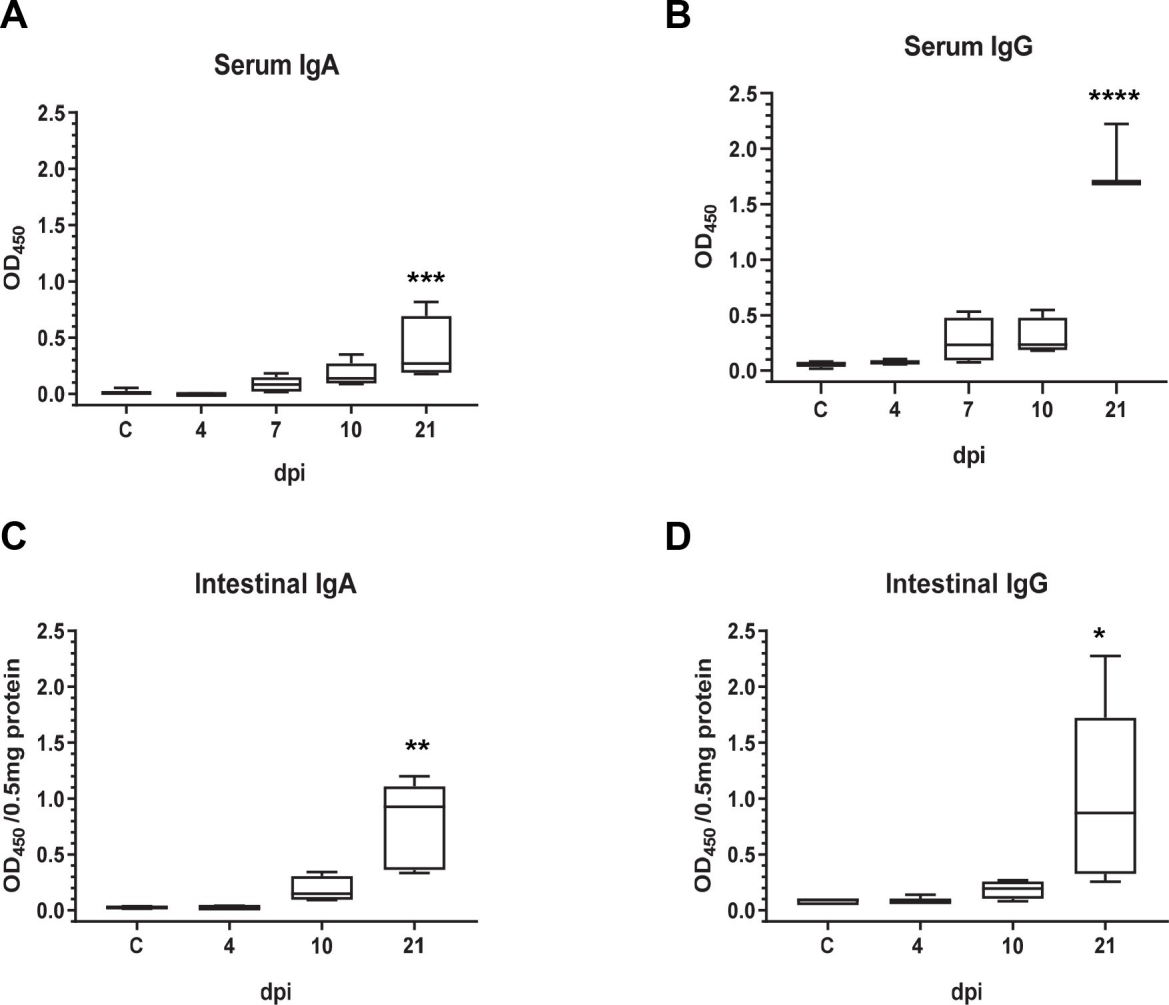
PEDV specific antibody response in serum and intestine of PEDV infected pigs. Serum (A and B) and intestinal homogenate (C and D) collected at indicated dpi were diluted 1:125. PEDV specific IgA and IgG in the serum were assessed by ELISA using purified PEDV coated microtiter plates and HRP conjugated goat anti pig IgA or goat anti pig IgG. OD_450_ values were normalized to 0.5mg of total protein in intestinal homogenate. C: uninfected control. *p<0.05, ** p <0.005, *** p <0.0005, and **** p <0.0001 (n = 3 to 5 pigs in each group).

At 7 dpi, PEDV specific IgG and IgA antibodies were detectable above background in serum but not at 4 dpi (Fig 3A and 3B). Similarly, the rise in PEDV specific antibody levels in intestinal homogenate was seen at 10 dpi, while it remained at background levels at 4 dpi (Fig 3C and 3D). PEDV specific antibody levels were significantly increased at 21 dpi, both in the intestine and serum.

**Fig 4:**
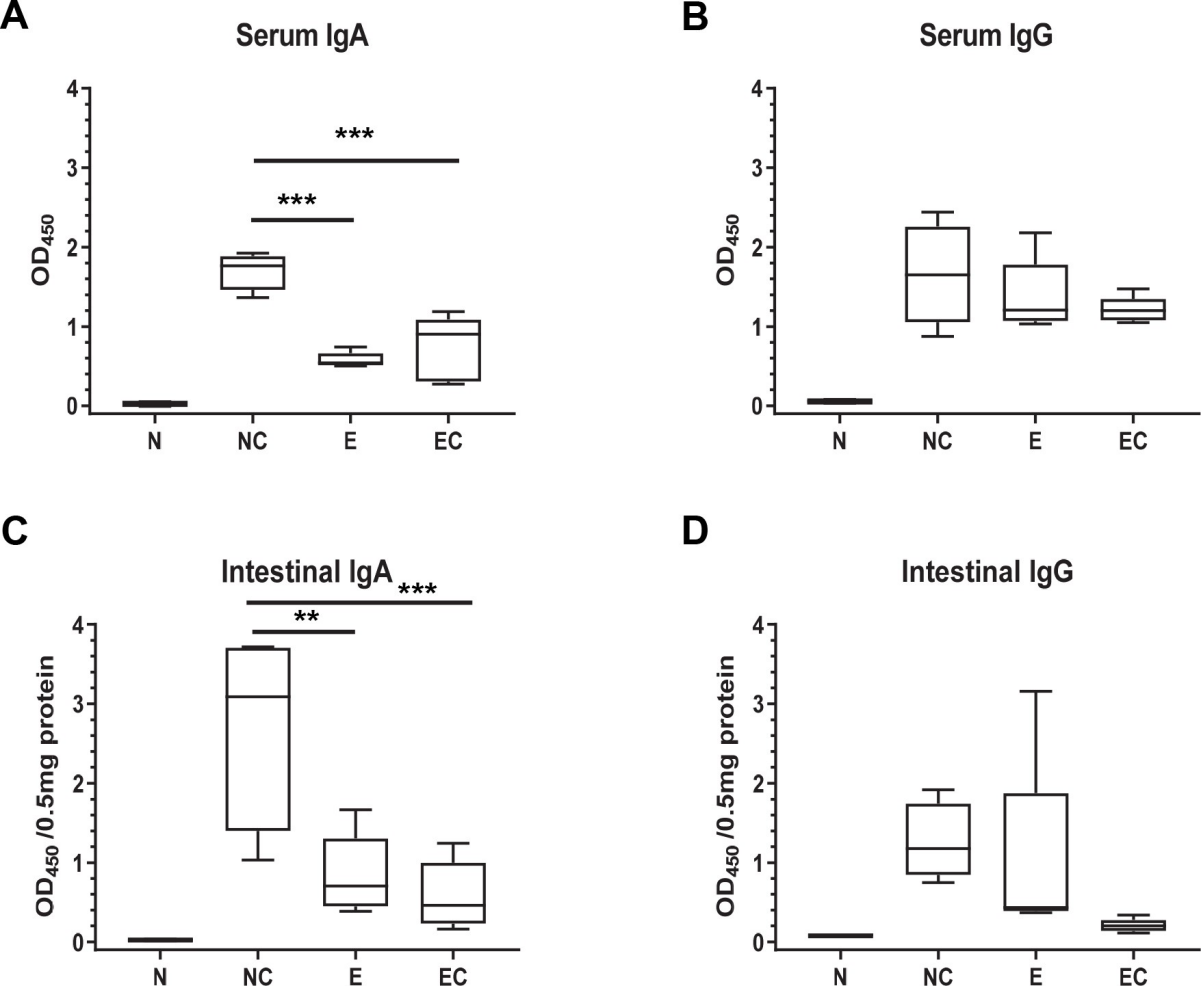
PEDV specific IgA and IgG antibody levels in pigs previously exposed to PEDV do not change significantly after challenge. Three-week-old pigs were exposed to PEDV. At 30 dpi, PEDV exposed pigs were challenged with a field isolate of PEDV. As a control, age matched naïve pigs were challenged with the same field isolate virus. 5 days post challenge tissues were harvested for analysis. OD_450_ values are normalized to 0.5mg of total protein in the intestine sample. N: naïve; NC: naïve, challenged; E: PEDV exposed; EC: PEDV exposed, challenged. ** p <0.005 and *** p <0.0005 (n = 3 to 5 pigs in each group).

To assess if challenge with PEDV resulted in an anamnestic response, serum and intestinal IgA and IgG levels were measured 5d post challenge. No significant increase in IgA or IgG antibody levels were observed in previously exposed pigs after challenge but remained higher than naïve control pigs (Fig 4). In contrast, naïve pigs with no previous PEDV exposure had significantly higher levels of PEDV specific IgA antibodies in serum and in the intestine post challenge (Fig 4A and 4C) while IgG antibody levels were higher than controls but not significantly different from animals with prior exposure (Fig 4B and 4D).

**Fig 5:**
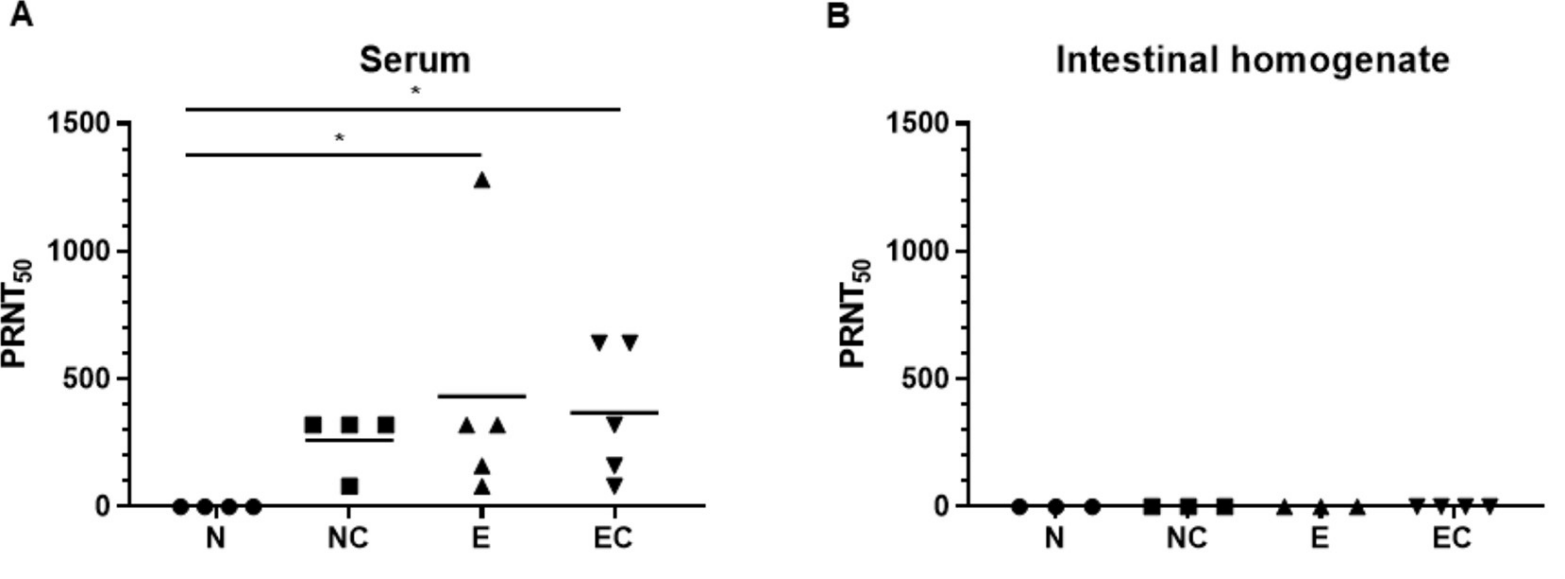
PEDV infected animals show high serum 50% plaque reduction neutralization titer (PRNT_50_). Serum (A) or intestinal homogenate (B) from naïve (N), naïve, challenged (NC), PEDV exposed (E), or PEDV exposed, challenged (EC) pigs were tested for their ability to neutralize PEDV by immunoplaque reduction assay. PRNT_50_: Reciprocal of the highest dilution of serum which showed ≥50% neutralization activity. *p<0.05 (n = 4 to 5 pigs n each group).

To determine if the antibodies generated had the ability to neutralize PEDV infectivity, PRNT_50_ from serum and intestinal homogenates collected at 5 dpc was determined using a virus-neutralizing assay. Serum samples from PEDV infected groups had significantly higher neutralization titer compared to naïve, uninfected animals (Fig 5A). However, no significant difference in virus neutralization were observed in serum of PEDV exposed animals and naïve animals after challenge. PEDV neutralizing activity was not detected in intestinal homogenate from animals in any of the groups (Fig 5B). Since only <20% reduction in number of plaques was observed with the lowest dilution of 1:4, we could not determine PRNT_50_ in intestinal homogenate samples.

### Phenotyping of immune cells in MLN and PBMCs

**Fig 6:**
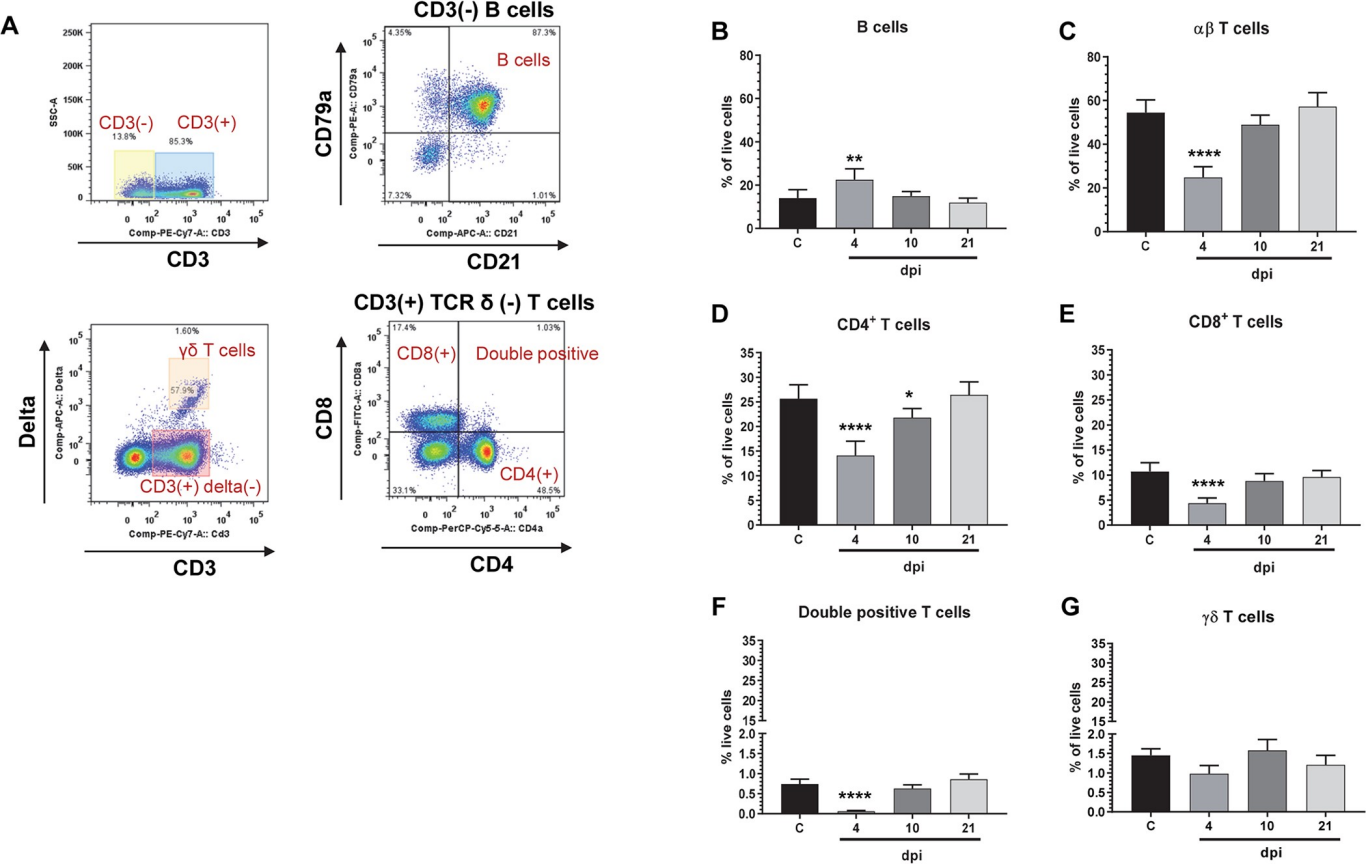
Frequency of B cells increase and T cells decrease in MLN at 4 dpi. Mononuclear cells isolated from MLN at indicated time dpi were analyzed by flowcytometry. (A) Flow cytometry gating to identify B cells (CD3- CD79a+ and CD21+ cells), αβ T cells (CD3+ and TCR δ-) and γδ T cells (CD3+ and TCR δ+). (B to G) Quantification of immune cells in MLN. C: uninfected control. * p <0.05, ** p <0.005, and **** p <0.0001 (n = 3 to 5 pigs in each group).

**Fig 7:**
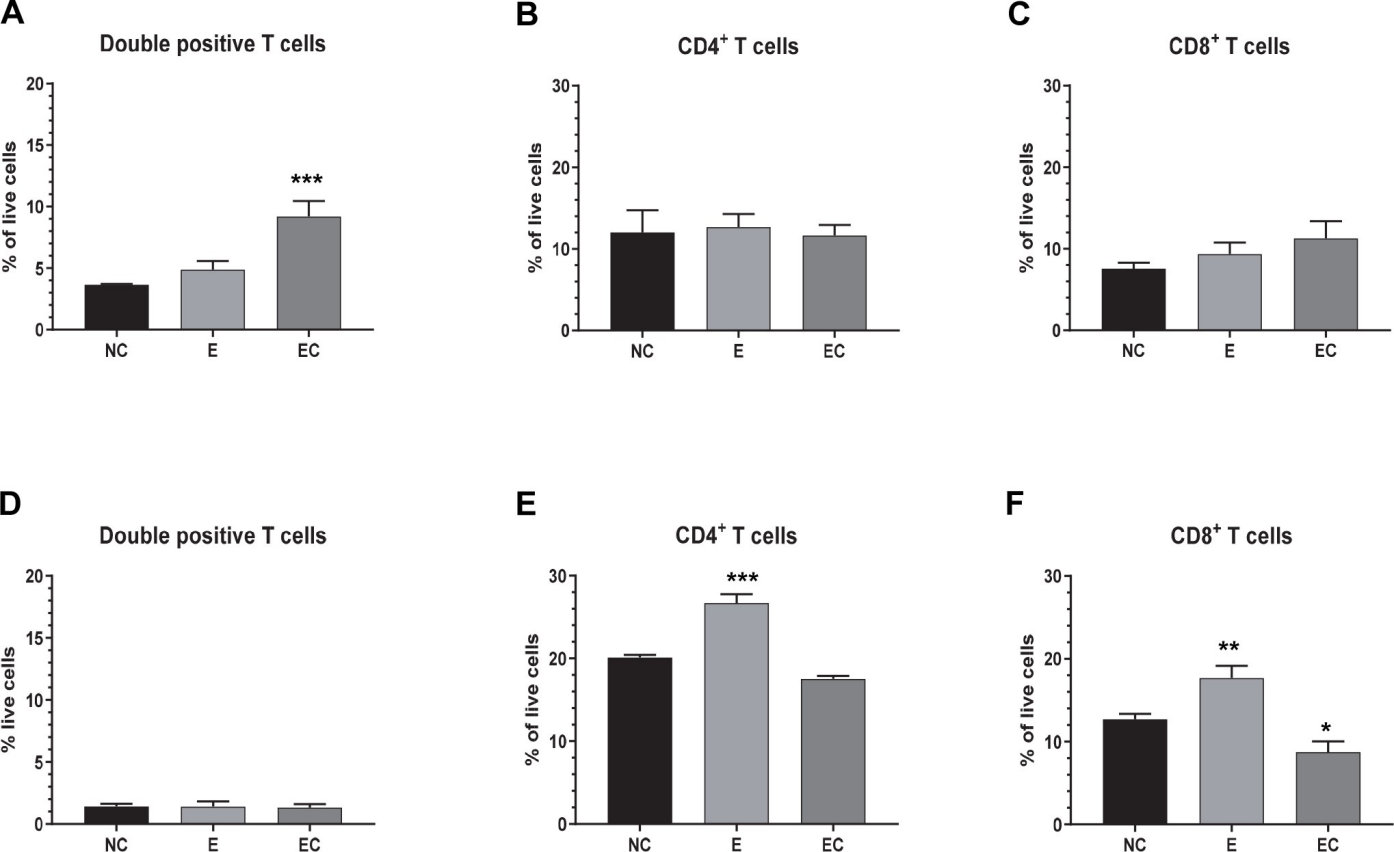
Frequency of CD4/CD8 double positive T cells increase in the MLN but not in blood after challenge. Mononuclear cells isolated from MLN (A-C) or peripheral blood (D-F) were analyzed by flowcytometry. NC: naïve, challenged; E: PEDV exposed; EC: PEDV exposed, challenged. * p <0.05, ** p <0.005, *** p <0.0005 (n = 3 to 5 in each group).

Flow cytometry analysis was performed to evaluate changes in the frequency of immune cell phenotypes within the mesenteric lymph node (MLN) and in the peripheral blood (PBMC). At 4 dpi PEDV-infected pigs had a significantly higher frequency of B cells and lower frequency of CD4, CD8 and CD4/CD8 double positive (DP) T cells in the MLN compared to uninfected pigs (Fig 6B–6F). These changes in the lymphocyte frequencies in the MLN returned to levels similar to uninfected animals by 10 and 21 dpi, albeit CD4+ T cell numbers were slightly lower at 10 dpi compared to uninfected. No significant change in frequency of γδ T cells were observed in MLN of PEDV infected pigs compared to control (Fig 6G). In contrast, when pigs with prior exposure to PEDV were challenged, frequency of DP T cells was significantly increased at 5 d post challenge in MLN compared to naïve- challenged pigs at the same time point (Fig 7A). However, there was no difference in frequency of either CD4+ or CD8+ T cells in the MLN (Fig 7B and 7C). This increase in DP T cells frequency in the MLN was not reflected in the blood (Fig 7D) post challenge. However, CD4+ and CD8+ (single positive) T cells in the peripheral blood were significantly higher in PEDV exposed pigs compared to challenged pigs (Fig 7E and 7F).

## Discussion

Understanding the immune responses against PEDV is important to prevent infection and to design better control strategies for PEDV. In the present study, we evaluated the immune response to PEDV in 3-week-old weaned piglets after infection with PEDV and assessed if prior exposure to virus protects against re-infection with virulent PEDV and induces a stronger local immune response in the GI tract. We found that pigs previously infected with PEDV are completely protected from virus shedding after re-infection and induced both humoral and cell mediated immune response with increase in PEDV specific IgA and IgG antibodies in intestine and serum. We also observed increase in frequency of CD4/CD8 double positive (DP) memory T cells in the MLN after challenge in pigs previously exposed to PEDV.

Humoral responses are essential for protection against PEDV infection in the neonatal pig. The first protection for the newborn piglet comes from the lactogenic immunity provided by its mother through colostrum and milk [9, 17]. Previous studies showed that sows previously exposed to mildly virulent strain of PEDV induce lactogenic immunity and cross protect piglets against virulent PEDV [18]. The immune response to PEDV is also determined by route of exposure [12, 15]. Sows vaccinated orally with live attenuated PEDV induced better passive protection to piglets compared to the same vaccine administered by intramuscular injection [15]. Several studies showed that mucosal immunity is induced effectively when vaccines are delivered at the mucosal surface [19], where microfold (M) cells take up and transport the antigens to dendritic cells and macrophages in the local lymph nodes to initiate antigen specific immune response. In addition, antigen specific response activated at a specific mucosal surface, such as intestine, can mediate an effector response at a distant site. Immune response initiated at the GI surface reaches the mammary gland via gut-mammary gland-sIgA axis and provides passive lactogenic immunity in suckling piglets [20, 21]. Recently it has been shown that induction of lactogenic immune response in pregnant sows depends on stage of gestation and sows infected with PEDV in second trimester provided 100% protection in suckling piglets [11]. While neonatal piglets are protected from PEDV by passive lactogenic immunity induced by vaccinating sows, these piglets become susceptible to PEDV post-weaning, making the induction of active immune responses an important protective mechanism in weaned piglets. Age resistance to clinical disease by PEDV has been described earlier and neonatal piglets exhibit severe clinical signs compared to weaned piglets [1, 22, 23]. In our study, only 50% of pigs exposed to PEDV showed mild diarrhea although all exposed pigs were infected with PEDV and showed virus shedding as determined by PEDV RT-qPCR on rectal swab samples. The duration of virus shedding depends on age of animal and infectious dose [23] and our results are consistent with previous reports, where the virus shedding was observed up to 18 to 28 days in three-week-old pigs [22–24]. Furthermore, PEDV exposed pigs were completely protected against re-infection when challenged 30 d after initial exposure. A similar trend was reported earlier in challenge studies where pigs previously exposed to virulent PEDV were shown to be protected from infection at challenge and absence of fecal viral shedding after second infection [10, 25].

PEDV infects and replicates in the enterocytes lining the small intestine [7]. We assessed the viral load within the intestinal mucosa by RT-qPCR. In agreement with the results of rectal swab samples, PEDV RNA was detected in the intestine of all infected pigs. Interestingly, presence of viral RNA was observed in the intestine even after fecal viral shedding was stopped in pigs exposed to PEDV (35 dpi), suggesting persistence of the virus in the animal without shedding. Similar results were observed in a previous study in 4-week-old pigs, where small intestine from 70% of pigs orally inoculated with USA/KS/2013 PEDV isolate were PCR positive at 42 dpi even though immunohistochemistry of intestinal tissue was negative for PEDV N protein and feces were PEDV negative by PCR [26]. This finding suggests that post weaning pigs may become reservoirs for PEDV, pose a risk for transmission and possible viral re-emergence. Further studies are required to examine how long-term viral RNA persist in the intestine after primary exposure.

Persistence of virus in lung and intestine up to 104 days after inoculation was reported previously in a related coronavirus, transmissible gastroenteritis virus (TGEV) [27]. In our present study, intestinal viral RNA load did not increase after PEDV challenge of previously exposed animals. In contrast, challenge infection in naïve pigs showed significantly higher levels of viral RNA in the feces and in intestinal tissue suggesting that prior exposure afforded protection from shedding and consequently transmission. Although the assessment of viral sequences could not distinguish between inoculum and challenge viruses, one could postulate that the inability to shed virus is likely due to a lack of replication of the challenge virus in intestines previously exposed to PEDV. More studies are needed to evaluate the implications of residual viral RNA detected in the intestine post PEDV infection.

Both systemic and mucosal immune responses play an essential role in protection against PEDV infection. In fact, mucosal IgA antibody response was shown to correlate with protection [10, 28]. In the present study, all pigs infected with PEDV seroconverted by 7 dpi with detectable levels of both virus specific IgG and IgA antibodies in serum. Serum antibody responses peaked at 21 dpi and continued to be elevated at 35 dpi. PEDV specific IgA and IgG antibodies followed a similar temporal pattern in intestinal homogenates of infected animals as in serum. Intestinal IgA antibodies were detected as early as 5 d after challenge in pigs with no previous exposure to virus. Interestingly, challenge with PEDV did not elicit an anamnestic humoral immune response either in the systemic or mucosal compartments.

Both IgA and IgG antibody levels in serum and intestinal homogenate were comparable in PEDV exposed animals with and without challenge. This finding is consistent with previous reports [10, 29] showing little or no increase in serum IgA and IgG titers or PEDV specific IgA and IgG antibody secreting cells in gut associated lymphoid tissue after challenge in pigs exposed to virulent PEDV. Interestingly, the antibody response to challenge virus in naïve pigs was higher than that was observed after initial infection with PEDV USA/Colorado/2013. While significant antibody response to PEDV was observed only at 21 dpi during primary infection with PEDV USA/Colorado/2013 (Fig 3), the challenge virus induced robust IgA antibody response as early as 5 dpi in pigs without previous PEDV exposure (Fig 4A–4D “I”). This could be due to differences in viral dose of inoculation during primary infection and challenge. Moreover, it is likely that the pathogenesis of viruses used for infection and challenge are different, given the field strain used in these experiments was not characterized. It has been shown that higher and more rapid PEDV specific IgA antibody responses were induced in virulent PEDV inoculated piglets compared to piglets inoculated with an attenuated S-INDEL strain [28]. Neutralizing antibodies in the gut may play an important role in intestinal mucosal immunity and prevent enteric virus infection like PEDV. Previous studies demonstrated presence of neutralizing antibodies in colostrum and milk of PEDV infected sows [30, 31]. In our study, virus neutralizing activity was observed only in serum but not in the intestinal homogenate of infected pigs. The intestinal homogenate used in this study represents a limited sample of the secreted fraction of the IgA antibody and may not be sufficient to assess the neutralizing capacity of the mucosal response to PEDV.

While importance of humoral immune response in PEDV infection is well studied, reports on cell mediated immune responses to PEDV infection in swine have been limited. In an earlier study, virus specific lymphoproliferative response was detected in MLN, blood, and spleen of pigs inoculated with virulent PEDV and showed a strong correlation with protection against virus challenge [32]. More recently, 5 d old suckling mice orally inoculated with PEDV were shown to have a higher frequency of CD8+ T cells but no significant difference in CD4+ T cells compared to control in peripheral blood at 14 dpi [12]. We demonstrate that the frequency of CD4+, CD8+, and CD4/CD8 DP T cells in the mesenteric lymph nodes decrease 4 d after PEDV exposure while B cells in the local secondary lymphoid tissue increase significantly. Like the antibody levels post challenge, anamnestic increase in T cell numbers in response to PEDV challenge was not detected in the blood or mesenteric lymph nodes.

Mature CD4/CD8 DP T cells have been defined in different species including human, mouse and swine [33]. In swine, they were characterized as memory T helper cells which are activated and proliferate by stimulation with recall viral antigen [34, 35]. Contrary to the response of CD4+ and CD8+ (single positive) T cells, CD4/CD8 DP T cells show a significant increase in frequency within the MLN after challenge in pigs previously exposed to PEDV compared to naïve animals following viral challenge. While this may reflect the expansion of PEDV specific effector memory cells, antigen specificity of these cells was not investigated in this study. The expansion of the DP T cell population in the local lymph node was not reflected in the peripheral circulation suggesting that this is a local response to the challenge virus. This finding indicates that prior exposure to PEDV will foster development of intestinal memory T cells that are capable of immunological response, and perhaps protection against reinfection. The lack of anamnestic responses in the humoral compartment of the mucosal response, argues for an important role of mucosal T cell memory response in PEDV. Further studies are needed to explore antigen specificity and function of the DP memory T cells.

In summary, we show that the immune response in piglets after primary exposure to PEDV results in protection against challenge with a virulent field isolate, in that it protects against viral shedding and may prevent virus transmission. In addition to antibodies present in the intestines after primary exposure, the increase of DP memory T cells in the mesenteric lymph nodes point to the importance of cell-mediated responses, in mediating protection to the piglet. Future studies may provide additional insights into understanding the immune response to PEDV and help in the design of future mucosal vaccines against PEDV infection.

## Supporting information

S1 Fig Optimization of PEDV specific IgA and IgG antibody ELISA. Microtiter wells were coated with purified PEDV USA/Colorado/2013 (PEDV Ag). As a negative control, similarly processed mock infected Vero 76 culture supernatant (Control Ag) was used. Serum from uninfected and PEDV infected pigs were used at 1:125 dilution for optimization of ELISA. (A) PEDV specific IgA was detected using HRP conjugated goat anti pig IgA (Bethyl Laboratories, Montgomery, TX; 1:10000 dilution). (B) PEDV specific IgG was detected using HRP conjugated goat anti pig IgG (Jackson ImmunoResearch, West Grove, PA; 1:3000 dilution). (C) To further validate specific binding of PEDV antibody in ELISA, mouse monoclonal antibody to PEDV spike protein (Clone S1D12, VMRD, Pullman, WA, 1:200 dilution) or mouse IgG2a isotype control were used as sample and detected using HRP conjugated goat anti-mouse IgG (R and D systems, Minneapolis, MN, 1:1000 dilution).
(TIF)
