# Supplementary figures and images for: Immune responses to porcine epidemic diarrhea virus (PEDV) in swine and protection against subsequent infection
Source: PLoS One. 2020 Apr 28;15(4):e0231723. doi: 10.1371/journal.pone.0231723 (PMC7188253; doi:10.1371/journal.pone.0231723)

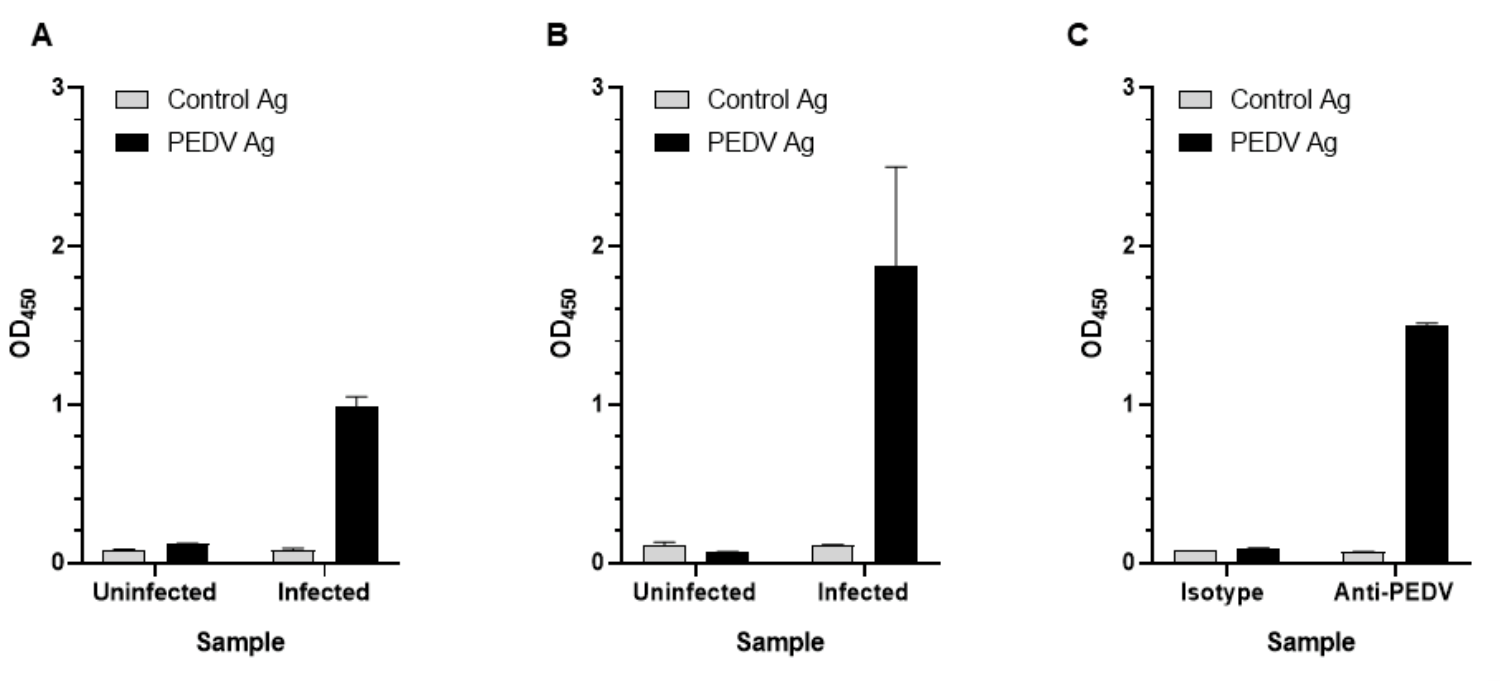

Supplement: S1 Fig — Microtiter wells were coated with purified PEDV USA/Colorado/2013 (PEDV Ag). As a negative control, similarly processed mock infected Vero 76 culture supernatant (Control Ag) was used. Serum from uninfected and PEDV infected pigs were used at 1:125 dilution for optimization of ELISA. (A) PEDV specific IgA was detected using HRP conjugated goat anti pig IgA (Bethyl Laboratories, Montgomery, TX; 1:10000 dilution). (B) PEDV specific IgG was detected using HRP conjugated goat anti pig IgG (Jackson ImmunoResearch, West Grove, PA; 1:3000 dilution). (C) To further validate specific binding of PEDV antibody in ELISA, mouse monoclonal antibody to PEDV spike protein (Clone S1D12, VMRD, Pullman, WA, 1:200 dilution) or mouse IgG2a isotype control were used as sample and detected using HRP conjugated goat anti-mouse IgG (R and D systems, Minneapolis, MN, 1:1000 dilution). (TIF) [file pone.0231723.s001.tif]
